# New England harbor seal H3N8 influenza virus retains avian-like receptor specificity

**DOI:** 10.1038/srep21428

**Published:** 2016-02-18

**Authors:** Islam T. M. Hussein, Florian Krammer, Eric Ma, Michael Estrin, Karthik Viswanathan, Nathan W. Stebbins, Devin S. Quinlan, Ram Sasisekharan, Jonathan Runstadler

**Affiliations:** 1 Department of Biological Engineering and Division of Comparative Medicine, Massachusetts Institute of Technology, Cambridge, MA, USA; 2 Koch Institute of Integrative Cancer Research, Massachusetts Institute of Technology, Cambridge, MA, USA; 3 Department of Microbiology, Icahn School of Medicine at Mount Sinai, New York, NY, USA.

## Abstract

An influenza H3N8 virus, carrying mammalian adaptation mutations, was isolated from New England harbor seals in 2011. We sought to assess the risk of its human transmissibility using two complementary approaches. First, we tested the binding of recombinant hemagglutinin (HA) proteins of seal H3N8 and human-adapted H3N2 viruses to respiratory tissues of humans and ferrets. For human tissues, we observed strong tendency of the seal H3 to bind to lung alveoli, which was in direct contrast to the human-adapted H3 that bound mainly to the trachea. This staining pattern was also consistent in ferrets, the primary animal model for human influenza pathogenesis. Second, we compared the binding of the recombinant HAs to a library of 610 glycans. In contrast to the human H3, which bound almost exclusively to α-2,6 sialylated glycans, the seal H3 bound preferentially to α-2,3 sialylated glycans. Additionally, the seal H3N8 virus replicated in human lung carcinoma cells. Our data suggest that the seal H3N8 virus has retained its avian-like receptor binding specificity, but could potentially establish infection in human lungs.

Influenza A viruses (IAVs) caused several pandemics in the past and continue to pose significant threats to human public health^1^. Wild migratory birds are the natural reservoirs of IAVs from which IAVs occasionally cross the species barrier to infect domestic birds, humans and several other mammalian species^2^. Marine mammals are particularly interesting hosts for IAVs. They are globally distributed and can migrate over long distances in the vicinity of coastal ecosystems and population centers, where they intersect with waterfowl and shorebirds in scenarios conducive to virus exchange^3^. Cases of IAV infection in marine mammals have been documented in the literature with several IAV subtypes including H1N1, H3N3, H3N8, H4N5, H4N6, H7N7 and H10N7^4^,^5^,^6^,^7^,^8^. The majority of these transmission events have implicated an avian source; however, serological evidence for seal infection by human H3 viruses has also been reported^9^,^10^,^11^,^12^. A spillover of an H7N7 seal virus to humans has been also described^4^,^13^. The wide variety of IAV strains infecting seals provides opportunities for genetic reassortment and/or adaptation, and it has been proposed that seals might play a similar role to pigs as mixing vessels for avian and human viruses^14^. With the exponential increase in protected seal populations and urbanization of coastal cities, the seal-human interface is continuously expanding, which creates a suitable environment for viral zoonotic transmissions^15^,^16^. The recently isolated H3N8 (A/harbour seal/New Hampshire/179629/2011) virus from an outbreak in harbor seals (*Phoca vitulina*) in New England demonstrated naturally acquired polymerase mammalian adaptation mutations^17^, indicating that it is of interest for human public health.

The viral surface glycoprotein, hemagglutinin (HA), is a key player in mediating transmission of IAVs. HA recognizes glycans with terminal sialic acid (SA) residues linked to galactose (Gal) via either an α-2,3 or α-2,6 linkage^18^. Glycan receptor binding specificity of IAVs helps define their host range and tissue tropism^19^. It is widely accepted that avian viruses preferentially bind to SAα-2,3Gal, while human influenzas bind to SAα-2,6Gal receptors^20^,^21^. Mutations switching HA’s binding specificity to the α-2,6 SA linkage is likely an important step in establishing human transmissibility^22^,^23^, with the overall glycan topology playing a critical role in determining receptor binding and host tropism^24^.

As a step towards assessing the public health risks, this study provides a comprehensive assessment of the receptor-binding specificity of a recombinant seal H3N8 HA to physiological glycans displayed on human and ferret respiratory tissues, and to chemically synthesized glycan arrays. Seasonal human-adapted strains of influenza are known to bind to SAα-2,6Gal receptors^25^, and are thus an epidemiologically relevant control for our study. In contrast to the human-adapted H3 control (A/Wyoming/03/2003), our findings suggest that the seal H3N8 HA preferentially binds to SAα-2,3Gal receptors that are abundant on human lungs^26^. We also present evidence that seal H3N8 virus replicated in human lung carcinoma cells, highlighting the importance of continuous monitoring of influenza viruses circulating in seals for the early detection of strains with enhanced zoonotic potential.

## Results

### Recombinant HA protein expression and purification

**Figure 1:**
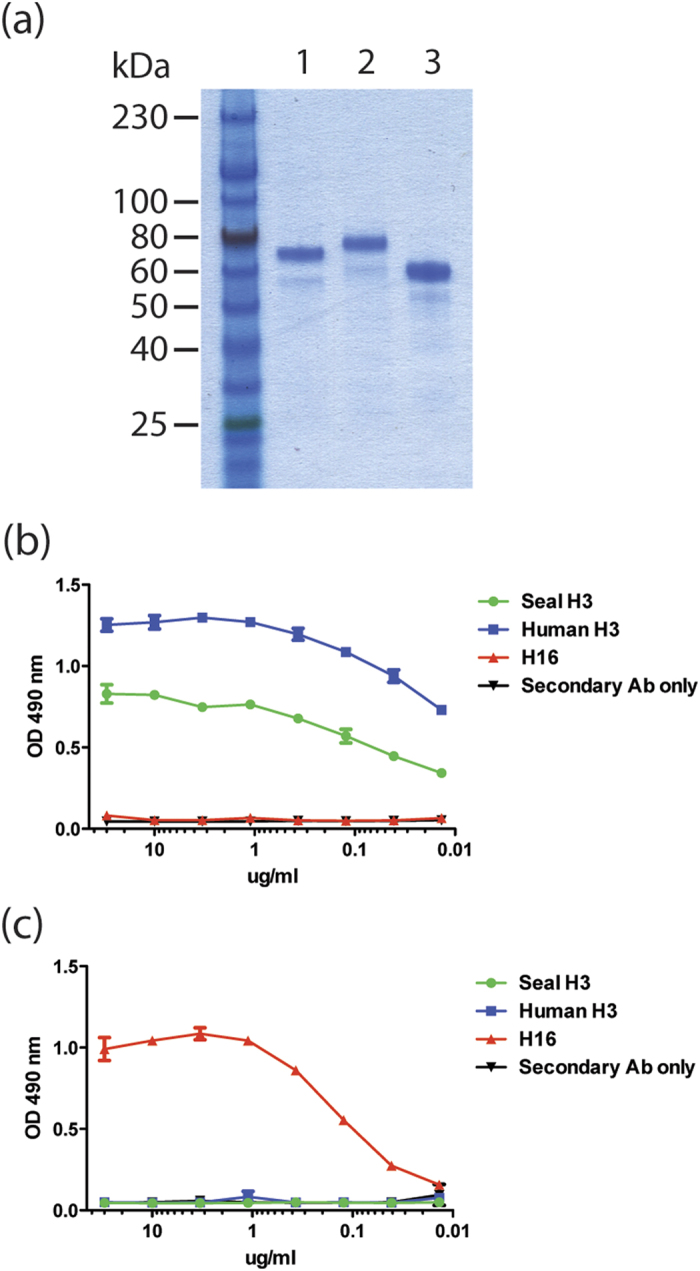
Expression and purification of recombinant HA proteins. (**a**) Coomassie blue stained SDS gel showing purified seal H3N8 HA (lane 1), human H3N2 HA (lane 2) and H16N3 (A/black headed gull/Sweden/5/1999) HA control (lane 3). (**b**) ELISA optical density values showing reactivity of the H3-specific monoclonal antibody 12D1 to recombinant seal H3 (green) and human H3 (blue) HA proteins. No reactivity was detected to the H16 control HA (red). (**c**) A control ELISA was performed using CR6261 antibody specific for group 1 HAs including H16, but not H3 subtype (which belongs to group 2). As expected CR6261 reacted with H16 HA (red), but showed no reactivity to the two H3 HAs.

Large amounts of soluble trimeric HA proteins were produced through expression in insect cells using the baculovirus system. This system has been successfully used before to produce biologically active recombinant HA for structural and biochemical studies^27^,^28^. The purity and identity of expressed proteins were assessed by SDS-PAGE and ELISA. As shown in Fig. 1(a), Coomassie blue stained gel shows purified recombinant seal H3, human H3 and H16 control HAs. To confirm the identity of the recombinant HAs, each was tested by direct coating ELISA, where an H3-specific antibody (12D1) was found to react with both H3 proteins, but not an unrelated H16 control (Fig. 1(b)). In addition, a group 1 HA-specific antibody (CR6261) did not react to either of the seal or human virus H3 proteins, which both belong to group 2 HA (Fig. 1(c)). These findings confirmed that the identity and purity of the recombinant HA proteins (Fig. 1(a)) produced by the baculovirus system were of the correct subtype.

### Recombinant seal H3N8 HA binds to human and ferret lung tissues

**Figure 2:**
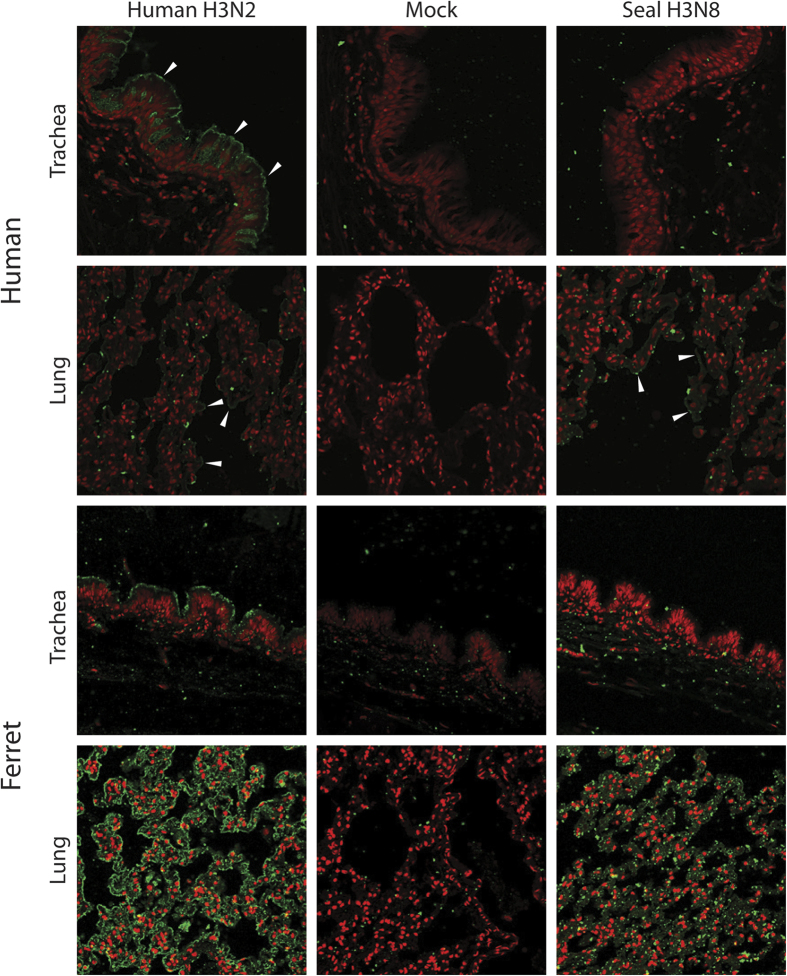
HA immunostaining of human respiratory tissues. Confocal microscopy images (20X) showing the varying binding affinity of human H3N2 and seal H3N8 HA proteins (green pointed by white arrows) to human and ferret tracheal and lung tissues (nuclei stained red).

**Figure 3:**
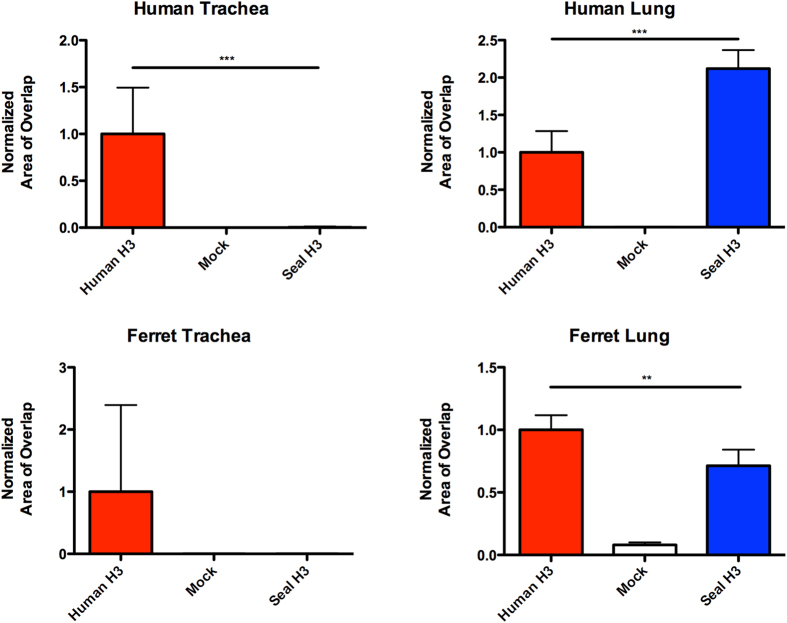
Quantitation of recombinant HA binding to human and ferret respiratory tissues. A minimum of three images were processed for each measurement. Error bars represent the 95% confidence intervals. Statistically significant differences between the human and seal H3 staining patterns across tested tissues are denoted by horizontal lines and asterisks.

In order to assess the ability of seal H3N8 virus to infect the upper respiratory tract of humans, which appears to be a requirement for efficient human-to-human transmission, we sought to investigate the binding patterns of its HA protein to physiological glycans present on human tissues. We compared the binding patterns of recombinant HA proteins derived from seal H3N8 against that of a seasonal human H3N2 virus (A/Wyoming/03/2003) to fixed human lung and tracheal tissue sections. HA proteins were allowed to bind to respiratory tissues, bound HA was detected by immuno-staining and the results were verified against negative control mock-stained tissue sections. Our results revealed that, in contrast to human H3N2, seal H3N8 HA exhibited minimal to no binding to the human trachea (Fig. 2). Nonetheless, it displayed greater binding affinity than the human H3N2 to human lung tissues. Quantifying the HA-specific signal revealed that human H3 bound efficiently to both human and ferret tracheas (p = 0.0007, 0.1250 respectively), whereas no binding signal could be quantified for the seal H3 on tracheal tissues. Both HA proteins showed binding to the cells lining the human and ferret lung alveolar tissues. Furthermore, the seal H3 bound stronger to human lungs than did the human H3, but the human H3 bound stronger to ferret lungs than did the seal H3 (Fig. 3, p = 0.0007, 0.0029 respectively).

### Recombinant seal H3N8 HA binds to α-2,3 sialylated glycans

**Figure 4:**
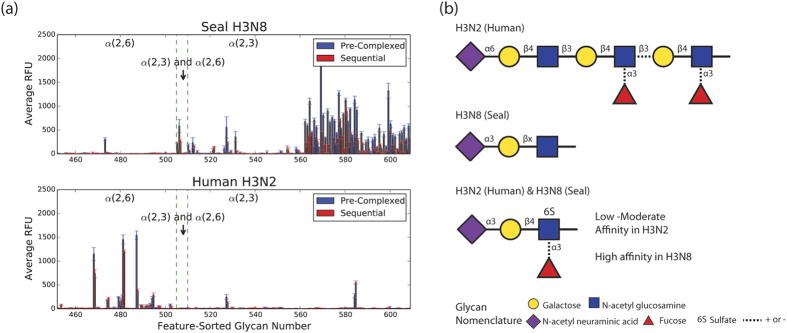
Glycan binding profiles of human H3N2 and seal H3N8 recombinant HA proteins. (**a**) The glycans on our array were sorted according to the type of their sialic acid linkages (X-axis) and plotted against the averaged relative fluorescence unit (RFU) values (Y-axis). Error bars represent SEM of 4 RFU values for each glycan tested in our array. (**b**) Glycan cartoons representing the most prevalent motif bound by either H3N2 HA (top), H3N8 HA (middle) or both H3N2 and H3N8 HA (bottom). Dotted lines indicate mixed presence and absence among commonly bound glycan motifs.

To obtain a more detailed and comprehensive picture of the receptor binding patterns of seal H3N8 HA, we tested its binding to an array of 610 glycans. Seal H3N8 virus harbored polymerase mutations that were indicative of mammalian adaptation^17^, which raised some concerns that this virus could potentially infect and efficiently spread in humans or other mammalian species. Therefore, we compared the seal HA binding pattern to that of the human-adapted H3N2 strain. Two methods were used for testing HA binding, one where the protein sample, primary antibody and secondary antibody were added sequentially to the slide, and the second where all reagents were pre-complexed together in a tube before adding to the slide. A high degree of overlap was observed for the glycan hits detected by both methods (Fig. 4(a)). The pre-complexing method has shown improved binding and slightly elevated fluorescence signals (relative fluorescence units or RFU) than when reagents were added sequentially (Fig. 4(a)). The elevated signal obtained with the pre-complexing method could be due to the increased number of antibody linked binding sites, which would result in a higher avidity to bind to HA. Supplementary Tables S1–S4 show all glycan hits (with p-values of less than 0.01) identified in our study for both HAs tested. One glycan, Neu5Ac α2–3Galb1–4(Fuca1–3)(6S)GlcNAcb, abbreviated as 6-sulfo sialyl Lewis X (Su-SLe^x^), was a common target for both HA proteins. Our glycan microarray screening (Fig. 4(a)) revealed that the H3N8 HA primarily binds to α-2,3 sialylated glycans similar to most avian adapted HAs^29^. Conversely, the human H3N2 HA binds predominantly to α-2,6 sialylated glycans, although some binding to α-2,3 sialylated glycans was also observed, which is consistent with previous studies^29^.

Using a qualitative assessment of the glycan structural features shared among the statistically significant hits from the array (Fig. 4(b)), we found that the human-adapted HA showed preferences for α-2,6 sialosides with >2 Gal-GlcNAc extensions (herein denoted long α-2,6 sialosides). Additionally, several of the top hits contained α-1,3 fucosylated GlcNAc residues on the second or third GlcNAc (relative to the penultimate sialic acid). The presence of a fucosylated GlcNAc had a relatively minor impact on H3N2 binding. In contrast, the seal H3N8 HA largely bound α-2,3-linked sialosides, with only one Gal-GlcNAc repeat (herein denoted short α-2,3 sialosides). Some modifications, such as 6-O-sulfation and fucosylation, were observed branching off of the first GlcNAc (relative to the penultimate sialic acid), however, these had minor effects on HA binding.

### Seal H3N8 viral growth kinetics

**Figure 5:**
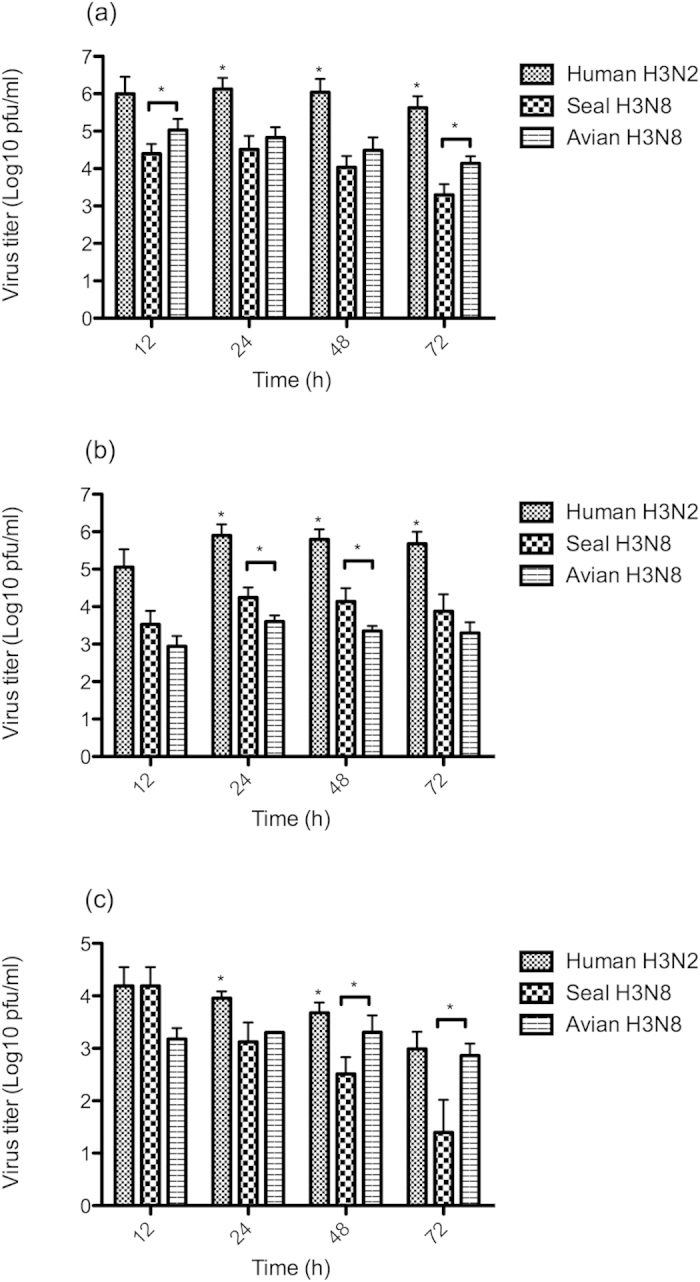
Replication kinetics of human H3N2, seal H3N8 and avian H3N8 viruses in MDCK (**a**), A549 (**b**) and DEF (**c**) cells. Cell monolayers seeded in 12-well plates were infected in duplicate, then allowed to adsorb for 60 minutes, then the virus inoculum were aspirated and cells were washed with PBS. Viral supernatants were collected at 12, 24, 48 and 72 hours post-infection and titrated by plaque assay in MDCK cells. Error bars represent the 95% confidence intervals (CI) of the mean of two independent experiments. Human H3N2 virus titers that were significantly higher than both of its seal and avian H3N8 counterparts are denoted by an asterisk (*). Statistically significant differences between the seal and avian H3N8 viruses are denoted by a horizontal line and an asterisk at the specified time points.

We examined the replication efficiency of seal H3N8, human H3N2 (A/Brisbane/10/2007) and avian H3N8 (A/American green-winged teal/Interior Alaska/10BM07649R0/2010) viruses in three types of cells: Madin-Darby Canine Kidney (MDCK) and human lung carcinoma cells (A549), and an avian cell line: duck embryo fibroblasts (DEF). Cell monolayers were infected in duplicate at a multiplicity of infection (MOI) of 1, and viral titers in the supernatants were monitored over a period of 72 hours (h). All three viruses replicated efficiently in MDCK cells, however human H3N2 virus titers were significantly higher than the seal and avian H3N8 that replicated to comparable levels, particularly at 24 and 48 h post-infection (Fig. 5(a)). A similar pattern was observed for the human H3N2 virus in A549 cells (Fig. 5(b)), where it exhibited titers that were about two orders of magnitude higher than the other two viruses. Interestingly, the seal H3N8 virus titers were significantly higher than that of the avian H3N8 virus at 24 and 48 h post-infection (p = 0.0294). The poorest replication kinetics for all three viruses were observed in DEF cells, where the seal H3N8 virus displayed significantly lower titers than its avian counterpart, particularly at 48 h (p = 0.0286) and 72 h (p = 0.0265) post-infection (Fig. 5(c)).

## Discussion

In nature, influenza viruses inhabit the guts of wild birds primarily belonging to the orders Anseriformes and Charadriformes. In these birds, infection is generally clinically asymptomatic and the virus replicates mainly in the intestinal tract. Occasionally, these viruses acquire mutations that allow them to switch hosts and infect domestic birds and mammals, where they can replicate in the respiratory tract or other tissues, causing mild to severe disease symptoms^2^. This report is an in depth assessment of the receptor binding specificity of the seal H3N8 virus (A/harbour seal/New Hampshire/179629/2011) that emerged, most likely from avian origins, in the New England harbor seal population in late 2011^17^. Based on the differential ability to agglutinate guinea pig and swine red blood cells and co-staining of viral HA and SAα-2,6- positive seal respiratory epithelium, Anthony *et al.* concluded that this virus was able to bind to both SAα-2,3 and SAα-2,6 receptors, a feature of avian viruses adapting to humans^19^. Another recent study reached similar conclusions based on a solid-phase binding assay that relied on only 2 types of biotinylated glycans (α**-**2,3′SL or α**-**2,6′SL)^30^. We therefore performed a series of experiments to assess in greater detail the ability of a recombinant HA of the seal H3N8 virus to bind to physiological glycans present on human and ferret tissues and to a large representative library of 610 chemically synthesized glycans on an array format. Previous studies have shown there is no difference in receptor binding specificity of recombinant HA proteins expressed in mammalian and insect cells^31^,^32^. Moreover, the physiological human respiratory glycans were shown to be well represented on the array produced by the Consortium of Functional Glycomics used in our experiments^18^. Therefore, we are confident that our recombinant HA binding studies reflects the behavior of a native viral HA. Contrary to an earlier study^30^, our data suggested that seal H3N8 recombinant HA has retained its avian receptor binding specificity as physiologically relevant immunohistochemistry demonstrated binding to human lung, but not the tracheal tissues, and as seal H3N8 recombinant HA showed strong preference for SAα-2,3Gal receptors on a mammalian glycan array (Figs 2 and 3). These findings are in agreement with the recently published HA binding data that relied on an array composed of a smaller group of 96 glycans^33^ in which the binding patterns of a whole seal H3N8 virus were generally consistent with the recombinant HA protein used in our experiment. As expected, the HA of our human-adapted H3N2 (A/Wyoming/03/03) positive control showed strong binding to human trachea and moderate binding to alveolar tissues, which is consistent with previously published tissue staining data^26^,^34^. We also observed a similar staining pattern in ferret respiratory tissues, where the human H3N2 HA, unlike its seal H3N8 counterpart, bound to ferret tracheal tissue sections. This is consistent with the previous observations that the glycan receptor distribution on the human and ferret respiratory tissues is similar^35^,^36^, though not identical^37^.

Although it is widely accepted that human-adapted and avian strains prefer SAα-2,6 receptors and SAα-2,3Gal respectively, this correlation is not absolute for all influenza virus subtypes^38^. Binding to SAα-2,3Gal receptors did not restrict the avian-to-human transmission of human H5N1 (A/HK/156/97) viruses isolated from the 1997 Hong Kong outbreak^39^. Glycan array analysis of the highly pathogenic H5N1 (A/Vietnam/1203/2004) virus, which was isolated from a Vietnamese bird flu victim, revealed a binding preference for sialylated glycans with SAα-2,3 linkage^40^. Clinically, H5N1 infection in humans is characterized by lung involvement, where virus replication mainly takes place^41^. Furthermore, immuno-staining studies of an H5N1 virus (A/Vietnam/1194/04) revealed strong binding to type II pneumocytes of human lungs^42^. These findings indicated that highly pathogenic IAVs retaining avian receptor binding affinity could replicate and cause fatal disease in humans without a significant change in its receptor-binding affinity. Here we show that the seal H3N8 virus replicated more efficiently in human lung A549 carcinoma cells than its avian counterpart (Fig. 5(b)), suggesting that it could potentially establish infection in the lower respiratory tract of humans. However, since alterations of receptor binding preference seem to be a prerequisite for efficient human-to-human transmission^18^,^43^, the lack of α-2,6 sialylated glycan binding by seal H3N8 HA indicates that this virus has not yet acquired the mutations required for human adaptation and is unlikely to spread efficiently among humans. In their study of seal H3N8 virus airborne transmission, Karlsson and colleagues have detected an HA A134T mutation, known to alter the receptor-binding specificity of avian H5N1 viruses from α-2,3 to α-2,6 sialylated glycans, in viruses recovered from aerosol/droplet contact ferrets^30^. However, it is not clear whether this mutation facilitated aerosol transmission or if it emerged in the sentinel ferrets after transmission. In either case, combining with our results raises a question of whether the naturally acquired polymerase mutations (e.g. PB2 D701N) in the seal H3N8 virus may have more of an impact on ferret transmissibility than previously understood.

Additional features of cell surface glycans beyond the terminal sialic acid linkage were shown to be important in the binding of human-adapted versus avian-adapted HAs to their respective glycan receptors^44^. The breadth of the array used in our experiments, which is composed of 610 glycans, enabled us to also probe the structural determinants of the human versus seal H3 receptor binding. The long α-2,6 motif was found to be the most critical determinant of human-adapted HA binding. On the other hand, the seal H3N8 HA bound mainly to short α-2,3-linked sialosides, with only one Gal-GlcNAc repeat (Fig. 4(b)). These findings are supported by previous studies demonstrating that receptor topology governs specificity of human and avian receptors. The long α-2,6 sialosides are capable of adopting a flexible umbrella-like topology, and are the predominant receptor type for pandemic human-adapted HA. The short α-2,3 sialosides have been shown to adopt a cone-like topology, and are the predominant receptor type for avian-adapted HA^24^. Thus, we believe that the seal H3N8 shares receptor-binding characteristics similar to those of avian-adapted HAs. Interestingly, the fact that we could detect binding of both human and seal H3s to Su-SLe^x^ indicates that the seal H3N8 virus could be diverging away from its avian ancestors^17^. Enhanced binding to sulfated and/or fucosylated glycans with α-2,3 linkages, particularly Su-SLe^x^, was a common feature of IAVs isolated from terrestrial poultry, pigs and horses, but not duck viruses^45^. An earlier glycan array study has also shown that, in contrast to a duck H3 virus (A/Duck/Ukraine/1963), the HA of several human H1 and H3 viruses bound to Su-SLe^x^ 29.

In conclusion, seal H3N8 virus still maintains the avian-type receptor specificity, binds to human lung tissues and replicates in human lung carcinoma cells, which raises concerns about its potential to establish infection in the lower respiratory tract of humans. However, we believe that certain additional mutations will be required for this virus to gain human transmissibility. Data presented in this study coupled with the recently published seal H3N8 HA crystal structure^33^, could provide impetus for future studies using similar approaches to unravel mutations that could potentially facilitate binding to human receptors. This study also helps clarify our understanding of the circulation and adaptation of influenza virus in seals, which is needed for early detection and characterization of viruses with an enhanced potential to infect humans and to evaluate if marine mammal populations could be a reservoir for mammalian adaptation of potentially pandemic human influenza virus.

## Methods

### Viruses, cells and tissues

Seal H3N8 (A/harbour seal/New Hampshire/179629/2011) was obtained from Dr. Hon Ip (National Wildlife Health Center, Madison Wisconsin). Avian H3N8 (A/American green-winged teal/Interior Alaska/10BM07649R0/2010) was one of our own Alaskan isolates. Human H3N2 (A/Brisbane/10/2007) was obtained from Biodefense and Emerging Infections Research Resources Repository (BEI). Viral stocks were prepared by inoculating 10-day old embryonated chicken eggs and harvesting the allantoic fluid 3 days later. A549 lung carcinoma cells (ATCC CCL-185), DEF duck embryo fibroblasts (ATCC CCL-141) and MDCK cells (ATCC CCL-34) were maintained in DMEM containing 10% FBS and penicillin/streptomycin at a final concentration of 50 IU/ml penicillin and 50 μg/ml streptomycin. Formalin-fixed paraffin-embedded tissue sections of the human trachea and lung were purchased from BioChain. Archival normal ferret tissue specimens were kindly provided by the Histology Laboratory of MIT’s Division of Comparative Medicine. These tissues were fixed in 10% neutral buffered formalin for 24 hours and processed by routine paraffin embedding and sectioned at 4–6 μm for subsequent immuno-staining.

### Viral growth kinetics and titration

Twelve-well plates were seeded with MDCK, A549 or DEF cells and allowed to grow until confluent monolayers were obtained. On the day of the experiment, one monolayer from each type of cells was trypsinized in 0.25% Trypsin-EDTA and counted in a hemocytometer. Titrated viral stocks were diluted and used to infect cells in duplicates at a multiplicity of infection (MOI) of 1. Briefly, viruses were allowed to adsorb for 60 minutes (mn), then the virus inoculum were aspirated and cells were washed once with sterile PBS. Supernatants were collected at 0, 12, 24, 48 and 72 hours post-infection and titrated by plaque assay on fresh MDCK monolayers.

### Recombinant HA expression and purification

Recombinant HAs were expressed as described before^46^. Briefly, genes encoding the ectodomains of the A/Wyoming/03/03 and A/harbour seal/New Hampshire/179629/2011 HAs were cloned into a modified pFastBacDual (Invitrogen) baculovirus transfer vector that harbors a C-terminal T4 foldon trimerization domain, a thrombin cleavage site and a hexahistidine tag. The identity of the recombinant baculovirus vectors was verified by Sanger sequencing, which was carried out by the sequencing services of Macrogen. To generate recombinant bacmids, the transfer plasmids were transformed into DH10Bac competent bacteria (Invitrogen). Bacmids were then transfected into Sf9 insect cells to generate recombinant baculovirus. Cell supernatants were incubated with NiNTA resin (Qiagen) and protein preps were concentrated and buffer exchanged to pH 7.4 PBS using Amicon Ultra centrifugation columns (Millipore). Recombinant HA proteins were checked for structural integrity and identity using SDS-PAGE and ELISA as described before^47^.

### Immuno-staining of human and ferret respiratory tissues

Tissue staining was carried out as previously described^48^. Briefly, the paraffin coating was melted, and slides were then blocked with 1% BSA-PBS, followed by incubation with HA pre-complexes at a ratio of 4:2:1 [HA (seal H3N8, human H3N2, or mock): primary antibody (mouse anti-His from Abcam): Secondary antibody (Goat anti-mouse labeled with Alexa Fluor from Lifetech)]. Slides were then immersed in propidium iodide (Lifetech) at a final concentration 1:100, then washed and finally mounted in anti-fade reagent (Lifetech) for confocal imaging using Zeiss 700 laser scanning microscope.

### Image quantitation

We used the scikit-image Python package for image quantification^49^. Briefly, images of human H3 binding trachea were treated as positive controls, and mock-stained slides were treated as negative controls. The images were separated into their red and green channels. In our particular staining protocol, the nuclei, which represent cells, are not directly in contact with the HA protein. Therefore, instead of computing the amount of red-green overlap, we sought to quantify the amount of green (protein) associated within an area around the red (cells) or vice versa (Figs S1 and S2). To identify regions of significant red (nuclei) or green (HA protein), we first applied an intensity threshold computed based on the positive control images, by using Otsu’s method^50^. We then sought to delineate a region around the nuclei or the HA protein boundaries. This was accomplished by computing the entropy of the thresholded red and green channels within a radius of 15 and 10 pixels respectively, and then thresholding the resulting image, identifying regions of significant entropy. A demonstration of this procedure is provided as an IPython HTML notebook on Github (supplementary materials). We then computed the number of pixels overlapping between the nuclei boundary regions and the HA protein regions. The threshold values computed for the positive control slides were averaged, and this value was also used for the negative control and seal H3 samples.

### Glycan array screening

Receptor binding specificities of seal H3N8 and human H3N2 HA recombinant proteins were tested on an array comprising 610 glycan targets. A list of the glycans used in this study (array version 5.1) can be found here: http://www.functionalglycomics.org/static/consortium/resources/resourcecoreh8.shtml. This array was manufactured by the Consortium for Functional Glycomics (CFG)^51^. Each protein was tested in 6 replicates at a concentration of 200 μg/ml in a binding buffer (20mM Tris-HCL pH 7.4, 150 mM sodium chloride, 2mM calcium chloride, 2mM magnesium chloride, 0.05% Tween 20 and 1% BSA). HA binding was tested by two methods, one where the protein sample, mouse anti-His primary antibody (Abcam) and Alexa-labeled anti-mouse secondary antibody (provided by CFG) were added in sequential steps to the slide, and another where all reagents were pre-complexed in a tube before adding to the slide. Each protein was tested in 6 replicates. The highest and lowest point from each set of 6 replicates was excluded to eliminate some of the false hits. The remaining 4 relative fluorescence unit (RFU) values were averaged and plotted for each protein (Fig. 4(a)). To identify the preferred receptor-binding motif of the seal and human subtype 3 hemagglutinin proteins (H3s), we analyzed the ‘hits’ identified on the glycan array. We assessed four key features: i) terminal sialic acid linkage (α-2,3 or α-2,6), ii) number of Hex-HexNAc repeats (n = 1 or n > 1; predominantly Gal-GlcNAc), iii) sulfation, and iv) fucosylation. These features were chosen because they were previously identified as determinants of hemagglutinin binding in human or avian adapted viruses^24^,^45^. In Tables S1–S4, the presence of a feature was indicated with a one and the absence with a zero. In certain cases, such as (Hex-HexNAc)_n_ repeats where n > 1, the exact number of repeats (n) was indicated in brackets next to the 1. In cases where two or more structurally identical branches were attached to the N-linked glycan ‘core’ (Man_3_GlcNAc_2_) or a GalNAc core, the number of branches was noted but only one branch was taken into account when assessing structural features. A qualitative assessment of the features was performed across the top binders, and a representative cartoon was drawn to depict common features of the glycans that bound each HA (Fig. 4(b)).

### Statistical analysis

For the glycan array experiments, we used the data to estimate a baseline value for non-binders. As we expect the integer values of the RFU to be distributed continuously at the non-binding baseline, and positive hits to be “broken” off from this continuity, we took the value after the first “break” in continuity as the estimated value for non-binding baseline. This was done as opposed to picking an arbitrary value to use across all data sets, in order to account for variability between each experiment. We computed a t-score, under the null hypothesis of non-binding using the estimated non-binding baseline value, and computed the corresponding p-value using a one-tailed t-test with 3 degrees of freedom. We then selected hits that had a p-value of less than 0.01 (supplementary Tables 1–4). For image quantitation, the Mann-Whitney U-test was used for comparisons between the human H3 and seal H3 on the lung and tracheal tissues of human and ferret (Fig. 3). Because the seal H3 binding values were all zero on the ferret trachea, the Wilcoxon signed rank test was used instead. For virus replication kinetics, the paired t-test was used for reported comparisons (Fig. 5).

## Additional Information

**How to cite this article**: Hussein, I. T. M. *et al.* New England harbor seal H3N8 influenza virus retains avian-like receptor specificity. *Sci. Rep.* **6**, 21428; doi: 10.1038/srep21428 (2016).

## Supplementary Material

Supplementary Text
