# Supplementary material for: New England harbor seal H3N8 influenza virus retains avian-like receptor specificity
Source: Sci Rep. 2016 Feb 18;6:21428. doi: 10.1038/srep21428 (PMC4757820; doi:10.1038/srep21428)
Supplement: Supplementary Text [file srep21428-s1.pdf]

Supplementary material for:

New England harbor seal H3N8 influenza virus retains avian-like receptor specificity and replicates in human lung cells

Islam T. M. Hussein[1], Florian Krammer[3], Eric Ma[1], Michael Estrin[1], Karthik Viswanathan[2], Nathan W. Stebbins[1,2], Devin S. Quinlan[1,2], Ram Sasisekharan[1.2], and Jonathan Runstadler[1*]

Department of Biological Engineering, Division of Comparative Medicine, Massachusetts Institute of Technology, Cambridge, MA, USA[1]; Koch Institute of Integrative Cancer Research, Massachusetts Institute of Technology, Cambridge, MA, USA[2]; Department of Microbiology, Icahn School of Medicine at Mount Sinai, New York, NY, USA[3]

Source code repository location and software dependencies for image quantitation:

Source code for image quantification was written using a combination of custom Python scripts and IPython notebooks. The Python packages required include:

- scikit-image 0.9.3 and all of its dependencies,
- pandas 0.13.1 and all of its dependencies,
- IPython 2.1 and all of its dependencies.

We used Python 2.7.6 for all of our analysis. Instructions for running the IPython environment are not provided here, as sufficient tutorials are abound online.

The notebooks and their contents are described below:

- Notebook "0. Functions Tutorial.ipynb" illustrates how the image quantification is performed using scikit-image functions.
- Notebooks "1. Identify positive control threshold values.ipynb" and "2. Process all images.ipynb" are where the actual quantification is performed.

Source code is published on Github at https://github.com/runstadler-lab/Seal-H3N8-Image-Analysis.

Supporting information legends:

Fig. S1: Representative images showing the areas of overlap between the red and green channels for human tissues. On each image, the top half is tracheal tissue staining, and the bottom half is lung tissue staining. Left panels: HA protein from human H3; middle panels: mock (control) staining; right panels: seal H3. Immunofluorescence images show nuclei (red) and purified HA protein (green) staining. Overlap images show the pixels where a defined area around the nuclei (as described in the methods section) and HA proteins overlap (white regions).

Fig. S2: Representative images showing the areas of overlap between the red and green channels for ferret tissues. Panels are analogous to Fig. S1.

Table S1: A list of all glycan hits (with p-values of less than 0.01) identified in our array screen for the human H3 using the sequential method

Table S2: A list of all glycan hits (with p-values of less than 0.01) identified in our array screen for the human H3 using the pre-complexing method

Table S3: A list of all glycan hits (with p-values of less than 0.01) identified in our array screen for the seal H3 using the sequential method

Table S4: A list of all glycan hits (with p-values of less than 0.01) identified in our array screen for the seal H3 using the pre-complexing method

Supplementary Figure S1

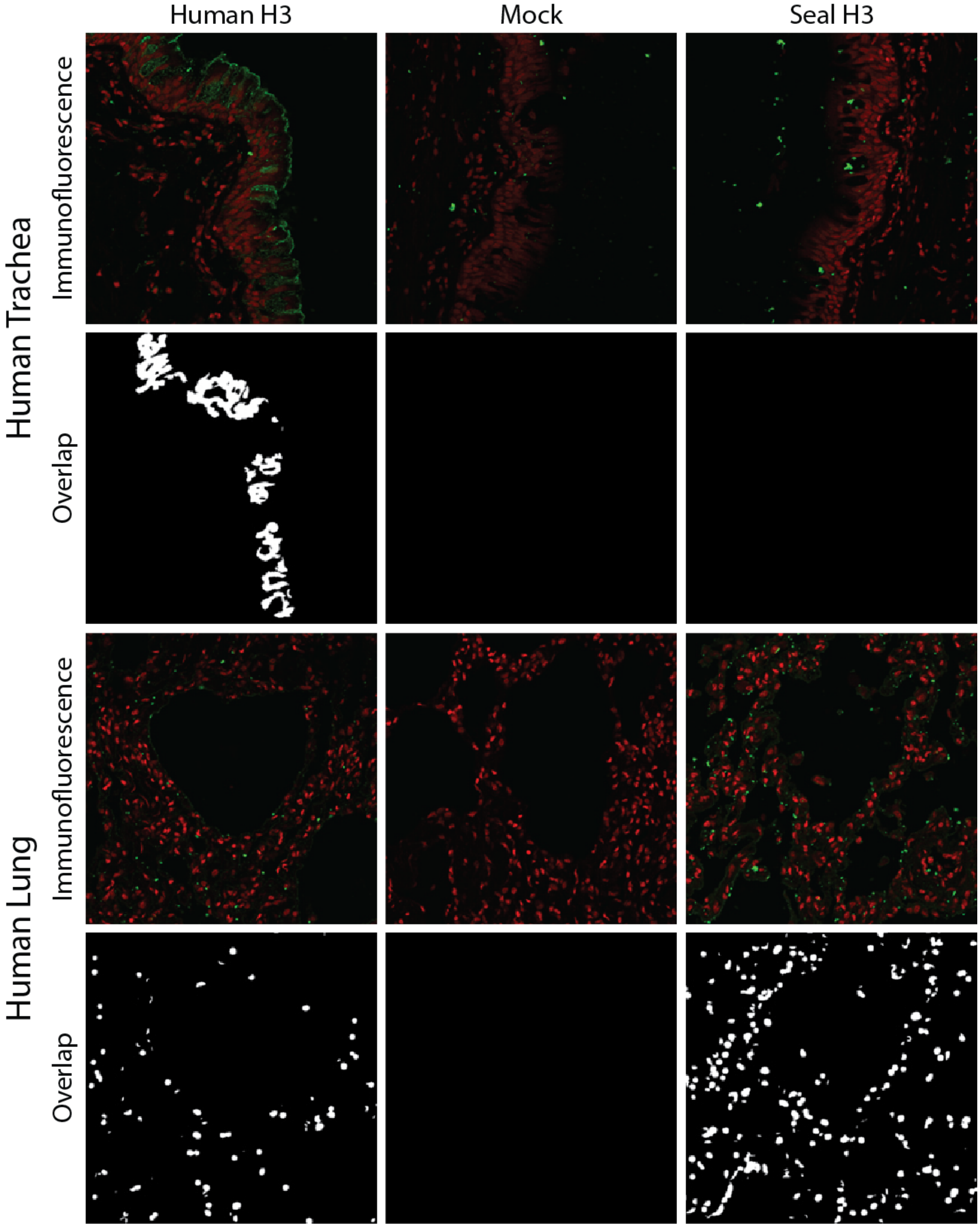

Supplementary Figure S2

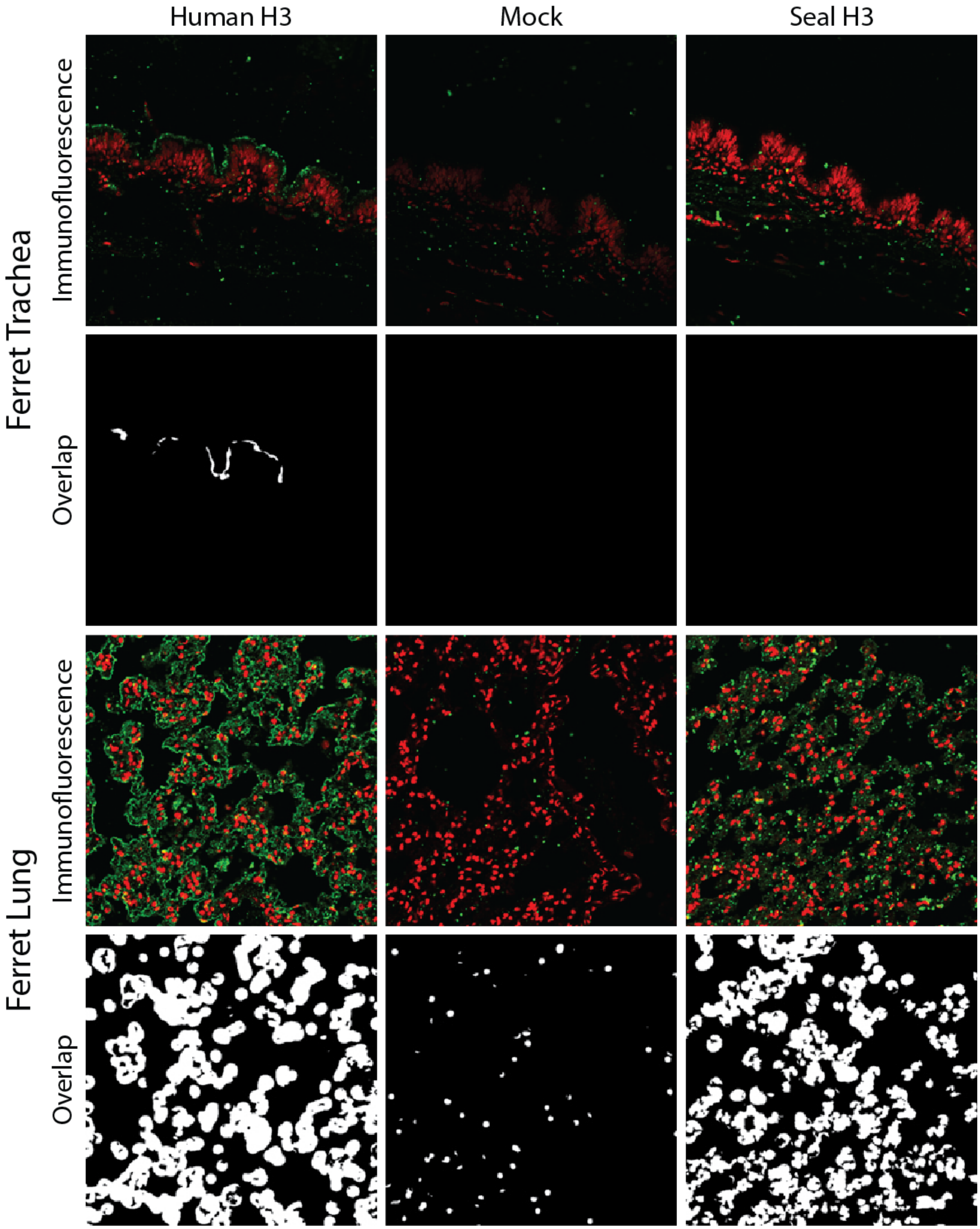

Table S1: A list of all glycan hits (with p-values of less than 0.01) identified in our array screen for the human H3 using the sequential method

Glycan #	Glycan name	Average RFU	StDev	% CV	t-value	p-value
332	Neu5Aca2-6Galb1-4GlcNAcb1-3Galb1-4GlcNAcb1-3Galb1-4GlcNAcb-Sp0	1208	28	2	42.5861448	1.4249E-05
606	Neu5Aca2-6Galb1-4GlcNAcb1-3Galb1-4GlcNAcb1-3Galb1-4GlcNAcb1-2Mana1-6(Neu5Aca2-6Galb1-4GlcNAcb1-3Galb1-4GlcNAcb1-3Galb1-4GlcNAcb1-2Mana1-3)Manb1-4GlcNAcb1-4GlcNAcb-Sp12	741	89	12	8.3686024	0.00178894
252	Neu5Aca2-3Galb1-4(Fuca1-3)(6S)GlcNAcb-Sp8	554	33	6	17.0374516	0.00022023
270	Neu5Aca2-6Galb1-4GlcNAcb1-3Galb1-4(Fuca1-3)GlcNAcb1-3Galb1-4(Fuca1-3)GlcNAcb-Sp0	385	33	9	11.6038849	0.00068729
271	Neu5Aca2-6Galb1-4GlcNAcb1-3Galb1-4GlcNAcb-Sp0	294	43	15	6.88087212	0.00314367
605	Neu5Aca2-6Galb1-4GlcNAcb1-3Galb1-4GlcNAcb1-6(Neu5Aca2-6Galb1-4GlcNAcb1-3Galb1-4GlcNAcb1-3)GalNAca-Sp14	222	20	9	11.1676948	0.0007694
393	GalNAca1-3(Fuca1-2)Galb1-3GalNAca1-3(Fuca1-2)Galb1-4GlcNAcb-Sp0	200	9	4	22.4104284	9.7272E-05
251	Neu5Aca2-3Galb1-4(6S)GlcNAcb-Sp8	133	16	12	8.20008119	0.00189762
601	Neu5Aca2-6Galb1-4GlcNAcb1-3Galb1-4GlcNAcb1-6(Galb1-3)GalNAca-Sp14	133	29	22	4.54224583	0.00999086
602	Neu5Aca2-6Galb1-4GlcNAcb1-6(Galb1-3)GalNAca-Sp14	85	15	17	5.76515903	0.00518632

Table S2: A list of all glycan hits (with p-values of less than 0.01) identified in our array screen for the human H3 using the pre-complexing method

Glycan #	Glycan name	Average RFU	StDev	% CV	t-value	p-value
270	Neu5Aca2-6Galb1-4GlcNAcb1-3Galb1-4(Fuca1-3)GlcNAcb1-3Galb1-4(Fuca1-3)GlcNAcb-Sp0	1545	89	6	17.4557169	0.00020489
332	Neu5Aca2-6Galb1-4GlcNAcb1-3Galb1-4GlcNAcb1-3Galb1-4GlcNAcb-Sp0	1452	100	7	14.5784082	0.00034995
606	Neu5Aca2-6Galb1-4GlcNAcb1-3Galb1-4GlcNAcb1-3Galb1-4GlcNAcb1-2Mana1-6(Neu5Aca2-6Galb1-4GlcNAcb1-3Galb1-4GlcNAcb1-3Galb1-4GlcNAcb1-2Mana1-3)Manb1-4GlcNAcb1-4GlcNAcb-Sp12	1151	136	12	8.46174265	0.00173239
252	Neu5Aca2-3Galb1-4(Fuca1-3)(6S)GlcNAcb-Sp8	279	46	17	6.01513825	0.00460359
251	Neu5Aca2-3Galb1-4(6S)GlcNAcb-Sp8	250	48	19	5.20513244	0.00689051
601	Neu5Aca2-6Galb1-4GlcNAcb1-3Galb1-4GlcNAcb1-6(Galb1-3)GalNAca-Sp14	248	11	4	23.570246	8.3665E-05
271	Neu5Aca2-6Galb1-4GlcNAcb1-3Galb1-4GlcNAcb-Sp0	226	30	13	7.62052922	0.00234529
605	Neu5Aca2-6Galb1-4GlcNAcb1-3Galb1-4GlcNAcb1-6(Neu5Aca2-6Galb1-4GlcNAcb1-3Galb1-4GlcNAcb1-3)GalNAca-Sp14	176	13	7	13.9921456	0.00039524
481	Neu5Aca2-6Galb1-4 GlcNAcb1-6(Neu5Aca2-6Galb1-4GlcNAcb1-3)GalNAca-Sp14	87	15	17	5.87927985	0.00490897
502	Neu5Aca2-6GalNAcb1-4(6S)GlcNAcb-Sp8	77	10	12	8.08584913	0.0019763
393	GalNAca1-3(Fuca1-2)Galb1-3GalNAca1-3(Fuca1-2)Galb1-4GlcNAcb-Sp0	76	18	24	4.19326671	0.01236847
55	Neu5Aca2-6Galb1-4GlcNAcb1-2Mana1-6(Neu5Aca2-6Galb1-4GlcNAcb1-2Mana1-3)Manb1-4GlcNAcb1-4GlcNAcb-Sp12	59	10	18	5.70878935	0.00533091

Table S3: A list of all glycan hits (with p-values of less than 0.01) identified in our array screen for the seal H3 using the sequential method

Glycan #	Glycan name	Average RFU	StDev	% CV	t-value	p-value
241	Neu5Aca2-3Galb1-4(Neu5Aca2-3Galb1-3)GlcNAcb-Sp8	887	78	9	11.3687944	0.00073001
474	Neu5Aca2-3Galb1-3GlcNAcb1-6(Neu5Aca2-3Galb1-3GlcNAcb1-2)Mana1-6(Neu5Aca2-3Galb1-3GlcNAcb1-2Mana1-3)Manb1-4GlcNAcb1-4GlcNAcb-Sp19	720	32	4	22.6667311	9.4024E-05
325	Neu5Aca2-3Galb1-4GlcNAcb1-2Mana1-6(Neu5Aca2-3Galb1-4GlcNAcb1-2Mana1-3)Manb1-4GlcNAcb1-4GlcNAcb-Sp12	442	49	11	9.10841969	0.00139824
471	Neu5Aca2-3Galb1-4GlcNAcb1-6(Neu5Aca2-3Galb1-4GlcNAcb1-3)GalNAca-Sp14	415	39	9	10.7365182	0.00086387
258	Neu5Aca2-3Galb1-4GlcNAcb1-3Galb1-4GlcNAcb1-3Galb1-4GlcNAcb-Sp0	409	40	10	10.3401205	0.00096479
252	Neu5Aca2-3Galb1-4(Fuca1-3)(6S)GlcNAcb-Sp8	396	44	11	8.91702011	0.00148751
257	Neu5Aca2-3Galb1-4(Fuca1-3)GlcNAcb1-3Galb1-4GlcNAcb-Sp8	345	29	8	11.7719198	0.00065876
240	Neu5Aca2-3Galb1-3(Fuca1-4)GlcNAcb1-3Galb1-4(Fuca1-3)GlcNAcb-Sp0	331	28	8	11.9713096	0.00062692
242	Neu5Aca2-3Galb1-3(6S)GalNAca-Sp8	311	67	22	4.62451086	0.0095186
331	Neu5Aca2-3Galb1-3(Fuca1-4)GlcNAcb1-3Galb1-3(Fuca1-4)GlcNAcb-Sp0	298	38	13	7.87660374	0.00213197
234	Neu5Aca2-3Galb1-3GalNAcb1-4(Neu5Aca2-3)Galb1-4Glcb-Sp0	283	56	20	5.07749635	0.00737808
462	Neu5Aca2-3Galb1-4GlcNAcb1-6(Neu5Aca2-3Galb1-4GlcNAcb1-2)Mana1-6(GlcNAcb1-4)(Neu5Aca2-3Galb1-4GlcNAcb1-4(Neu5Aca2-3Galb1-4GlcNAcb1-2)Mana1-3)Manb1-4GlcNAcb1-4GlcNAcb-Sp21	279	49	18	5.69981396	0.00535441
239	Neu5Aca2-3Galb1-3(Fuca1-4)GlcNAcb-Sp8	272	52	19	5.20510879	0.0068906
243	Neu5Aca2-6(Neu5Aca2-3Galb1-3)GalNAca-Sp8	266	35	13	7.50691175	0.00244899
261	Neu5Aca2-3Galb1-4GlcNAcb1-3Galb1-4GlcNAcb-Sp0	258	47	18	5.4424445	0.00609034
260	Neu5Aca2-3Galb1-4GlcNAcb-Sp8	243	14	6	16.8252088	0.00022859
251	Neu5Aca2-3Galb1-4(6S)GlcNAcb-Sp8	235	23	10	10.040618	0.00105163
264	Neu5Aca2-3Galb1-4Glcb-Sp8	234	44	19	5.32776318	0.00646094
259	Neu5Aca2-3Galb1-4GlcNAcb-Sp0	222	16	7	13.8160411	0.00041035
460	Neu5Aca2-3Galb1-4GlcNAcb1-4Mana1-6(GlcNAcb1-4)(Neu5Aca2-3Galb1-4GlcNAcb1-4(Neu5Aca2-3Galb1-4GlcNAcb1-2)Mana1-3)Manb1-4GlcNAcb1-4GlcNAcb-Sp21	210	15	7	13.6738143	0.00042313
263	Neu5Aca2-3Galb1-4Glcb-Sp0	209	46	22	4.54039086	0.01000185
255	Neu5Aca2-3Galb1-4(Fuca1-3)GlcNAcb-Sp8	194	43	22	4.46774413	0.01044509
534	Neu5Aca2-3Galb1-4(Fuca1-3)GlcNAcb1-2Mana-Sp0	193	56	29	3.48633866	0.01993606
604	GlcNAcb1-6(Neu5Aca2-3Galb1-3)GalNAca-Sp14	178	29	17	6.05739783	0.0045137
479	Neu5Aca2-3Galb1-4GlcNAcb1-6GalNAca-Sp14	165	26	16	6.44593893	0.00378598
247	Neu5Aca2-3Galb1-3GlcNAcb1-3Galb1-4GlcNAcb-Sp0	164	29	18	5.57986315	0.00568202
223	Neu5Aca2-3Galb1-3GalNAca-Sp8	148	36	24	4.12550768	0.01291245
393	GalNAca1-3(Fuca1-2)Galb1-3GalNAca1-3(Fuca1-2)Galb1-4GlcNAcb-Sp0	147	35	24	4.22994313	0.01208634
245	Neu5Aca2-3Galb-Sp8	144	16	11	9.01166287	0.00144245
238	Neu5Aca2-3Galb1-3(6S)GlcNAc-Sp8	92	14	15	6.7029754	0.00338759
526	Neu5Aca2-3Galb1-3GlcNAcb1-2Mana-Sp0	86	24	28	3.63263254	0.01796348
282	Neu5Gca2-3Galb1-4(Fuca1-3)GlcNAcb-Sp0	70	16	23	4.32734243	0.01137641
249	Neu5Aca2-3Galb1-3GlcNAcb-Sp0	64	5	8	11.8934885	0.0006391
596	Neu5Aca2-3Galb1-4GlcNAcb1-3Galb1-4GlcNAcb1-6(Neu5Aca2-3Galb1-4GlcNAcb1-3Galb1-4GlcNAcb1-3)GalNAca-Sp14	53	9	18	5.60638983	0.00560735
395	Gala1-3Galb1-3(Fuca1-4)GlcNAcb1-2Mana1-6(Gala1-3Galb1-3(Fuca1-4)GlcNAcb1-2Mana1-3)Manb1-4GlcNAcb1-4GlcNAc-Sp19	51	9	17	5.7547986	0.0052125
491	Neu5Aca2-3Galb1-3GlcNAcb1-6GalNAca-Sp14	48	6	12	8.69493248	0.00160081
461	Neu5Aca2-3Galb1-4GlcNAcb1-6(Neu5Aca2-3Galb1-4GlcNAcb1-2)Mana1-6(GlcNAcb1-4)(Neu5Aca2-3Galb1-4GlcNAcb1-2Mana1-3)Manb1-4GlcNAcb1-4GlcNAcb-Sp21	40	5	12	8.08110759	0.00197966

Table S4: A list of all glycan hits (with p-values of less than 0.01) identified in our array screen for the seal H3 using the pre-complexing method

Glycan #	Glycan name	Average RFU	StDev	% CV	t-value	p-value
317	Neu5Aca2-3Galb1-4GlcNAcb1-6(Neu5Aca2-3Galb1-3)GalNAca-Sp14	2060	112	5	18.3901594	0.00017542
325	Neu5Aca2-3Galb1-4GlcNAcb1-2Mana1-6(Neu5Aca2-3Galb1-4GlcNAcb1-2Mana1-3)Manb1-4GlcNAcb1-4GlcNAcb-Sp12	1326	159	12	8.31862329	0.0018203
474	Neu5Aca2-3Galb1-3GlcNAcb1-6(Neu5Aca2-3Galb1-3GlcNAcb1-2)Mana1-6(Neu5Aca2-3Galb1-3GlcNAcb1-2Mana1-3)Manb1-4GlcNAcb1-4GlcNAcb-Sp19	1278	47	4	27.2480309	5.4242E-05
252	Neu5Aca2-3Galb1-4(Fuca1-3)(6S)GlcNAcb-Sp8	1137	74	7	15.355428	0.00029996
241	Neu5Aca2-3Galb1-4(Neu5Aca2-3Galb1-3)GlcNAcb-Sp8	1136	34	3	33.8013306	2.8462E-05
471	Neu5Aca2-3Galb1-4GlcNAcb1-6(Neu5Aca2-3Galb1-4GlcNAcb1-3)GalNAca-Sp14	1102	66	6	16.81335	0.00022907
258	Neu5Aca2-3Galb1-4GlcNAcb1-3Galb1-4GlcNAcb1-3Galb1-4GlcNAcb-Sp0	946	23	2	42.0284069	1.4823E-05
462	Neu5Aca2-3Galb1-4GlcNAcb1-6(Neu5Aca2-3Galb1-4GlcNAcb1-2)Mana1-6(GlcNAcb1-4)(Neu5Aca2-3Galb1-4GlcNAcb1-4(Neu5Aca2-3Galb1-4GlcNAcb1-2)Mana1-3)Manb1-4GlcNAcb1-4GlcNAcb-Sp21	916	66	7	13.7836313	0.00041322
247	Neu5Aca2-3Galb1-3GlcNAcb1-3Galb1-4GlcNAcb-Sp0	902	45	5	20.2311528	0.000132
253	Neu5Aca2-3Galb1-4(Fuca1-3)GlcNAcb1-3Galb1-4(Fuca1-3)GlcNAcb1-3Galb1-4(Fuca1-3)GlcNAcb-Sp0	837	25	3	33.0067588	3.0563E-05
483	Neu5Aca2-3Galb1-4GlcNAcb1-2Mana1-6(Neu5Aca2-3Galb1-4GlcNAcb1-2Mana1-3)Manb1-4GlcNAcb1-4(Fuca1-6)GlcNAcb-Sp24	814	43	5	18.870182	0.00016246
331	Neu5Aca2-3Galb1-3(Fuca1-4)GlcNAcb1-3Galb1-3(Fuca1-4)GlcNAcb-Sp0	764	34	4	22.6495442	9.4237E-05
240	Neu5Aca2-3Galb1-3(Fuca1-4)GlcNAcb1-3Galb1-4(Fuca1-3)GlcNAcb-Sp0	712	41	6	17.3921281	0.00020713
460	Neu5Aca2-3Galb1-4GlcNAcb1-4Mana1-6(GlcNAcb1-4)(Neu5Aca2-3Galb1-4GlcNAcb1-4(Neu5Aca2-3Galb1-4GlcNAcb1-2)Mana1-3)Manb1-4GlcNAcb1-4GlcNAcb-Sp21	697	28	4	24.9037829	7.0979E-05
255	Neu5Aca2-3Galb1-4(Fuca1-3)GlcNAcb-Sp8	680	49	7	13.9234527	0.00040105
534	Neu5Aca2-3Galb1-4(Fuca1-3)GlcNAcb1-2Mana-Sp0	678	17	3	39.7076689	1.7572E-05
256	Neu5Aca2-3Galb1-4(Fuca1-3)GlcNAcb1-3Galb-Sp8	662	31	5	21.0415831	0.0001174
223	Neu5Aca2-3Galb1-3GalNAca-Sp8	626	70	11	8.92231826	0.00148494
242	Neu5Aca2-3Galb1-3(6S)GalNAca-Sp8	593	36	6	16.6701826	0.00023498
243	Neu5Aca2-6(Neu5Aca2-3Galb1-3)GalNAca-Sp8	587	137	23	4.27574547	0.01174556
234	Neu5Aca2-3Galb1-3GalNAcb1-4(Neu5Aca2-3)Galb1-4Glcb-Sp0	587	48	8	12.1114663	0.00060575
261	Neu5Aca2-3Galb1-4GlcNAcb1-3Galb1-4GlcNAcb-Sp0	566	33	6	17.3963678	0.00020698
251	Neu5Aca2-3Galb1-4(6S)GlcNAcb-Sp8	556	224	40	2.47870161	0.04468631
264	Neu5Aca2-3Galb1-4Glcb-Sp8	552	48	9	11.4950275	0.00070665
604	GlcNAcb1-6(Neu5Aca2-3Galb1-3)GalNAca-Sp14	539	79	15	6.8178467	0.00322735
257	Neu5Aca2-3Galb1-4(Fuca1-3)GlcNAcb1-3Galb1-4GlcNAcb-Sp8	528	19	4	28.0564279	4.9701E-05
294	Neu5Aca2-3Galb1-3GlcNAcb1-3Galb1-3GlcNAcb-Sp0	455	27	6	16.972784	0.00022273
461	Neu5Aca2-3Galb1-4GlcNAcb1-6(Neu5Aca2-3Galb1-4GlcNAcb1-2)Mana1-6(GlcNAcb1-4)(Neu5Aca2-3Galb1-4GlcNAcb1-2Mana1-3)Manb1-4GlcNAcb1-4GlcNAcb-Sp21	451	40	9	11.2096083	0.00076097
259	Neu5Aca2-3Galb1-4GlcNAcb-Sp0	436	34	8	12.8488605	0.00050869
239	Neu5Aca2-3Galb1-3(Fuca1-4)GlcNAcb-Sp8	435	17	4	25.7441802	6.4276E-05
260	Neu5Aca2-3Galb1-4GlcNAcb-Sp8	426	39	9	10.8200019	0.00084443
224	Neu5Aca2-3Galb1-3GalNAca-Sp14	413	55	13	7.48724511	0.00246755
246	Neu5Aca2-3Galb1-3GalNAcb1-3Gala1-4Galb1-4Glcb-Sp0	396	41	10	9.69303455	0.00116591
263	Neu5Aca2-3Galb1-4Glcb-Sp0	391	9	2	45.9511865	1.1345E-05
254	Neu5Aca2-3Galb1-4(Fuca1-3)GlcNAcb-Sp0	365	36	10	10.0799859	0.00103964
526	Neu5Aca2-3Galb1-3GlcNAcb1-2Mana-Sp0	358	57	16	6.31385821	0.0040148
249	Neu5Aca2-3Galb1-3GlcNAcb-Sp0	357	114	32	3.14382043	0.02575548
441	Neu5Aca2-3Galb1-4GlcNAcb1-3Galb-Sp8	338	18	5	19.1834905	0.00015468
272	Neu5Aca2-6Galb1-4Glcb-Sp0	308	31	10	10.0580109	0.00104631
250	Neu5Aca2-3Galb1-3GlcNAcb-Sp8	307	26	8	11.9473938	0.00063063
600	Neu5Aca2-3Galb1-4GlcNAcb1-3Galb1-4GlcNAcb1-6(Galb1-3)GalNAca-Sp14	303	53	18	5.69169235	0.0053758
238	Neu5Aca2-3Galb1-3(6S)GlcNAc-Sp8	296	48	16	6.17040844	0.00428437
295	Neu5Aca2-3Galb1-4GlcNAcb1-3Galb1-3GlcNAcb-Sp0	242	47	20	5.12142999	0.00720532
244	Neu5Aca2-6(Neu5Aca2-3Galb1-3)GalNAca-Sp14	226	16	7	13.8797182	0.0004048
11	Neu5Acb-Sp8	215	61	29	3.48982678	0.0198859
326	Neu5Aca2-3Galb1-4GlcNAcb1-2Mana1-6(Neu5Aca2-6Galb1-4GlcNAcb1-2Mana1-3)Manb1-4GlcNAcb1-4GlcNAcb-Sp12	190	22	12	8.5114841	0.00170315
596	Neu5Aca2-3Galb1-4GlcNAcb1-3Galb1-4GlcNAcb1-6(Neu5Aca2-3Galb1-4GlcNAcb1-3Galb1-4GlcNAcb1-3)GalNAca-Sp14	156	19	12	8.02053172	0.00202323
491	Neu5Aca2-3Galb1-3GlcNAcb1-6GalNAca-Sp14	143	22	15	6.52208437	0.00366181
459	Neu5Aca2-3Galb1-4GlcNAcb1-2Mana1-6(GlcNAcb1-4)(Neu5Aca2-3Galb1-4GlcNAcb1-2Mana1-3)Manb1-4GlcNAcb1-4GlcNAcb-Sp21	138	8	6	17.5037591	0.00020322
322	Neu5Aca2-8Neu5Aca2-8Neu5Acb-Sp8	124	46	37	2.66667911	0.03795246
592	Neu5Aca2-3Galb1-4GlcNAcb1-3Galb1-4GlcNAcb1-3GalNAca-Sp14	118	50	42	2.37829498	0.04888412
376	Neu5Aca2-3Galb1-4GlcNAcb1-3GalNAc-Sp14	111	32	29	3.45421496	0.02040568
329	Neu5,9Ac2a2-3Galb1-3GlcNAcb-Sp0	107	16	15	6.82865537	0.00321279
282	Neu5Gca2-3Galb1-4(Fuca1-3)GlcNAcb-Sp0	103	10	9	10.7122484	0.00086964
393	GalNAca1-3(Fuca1-2)Galb1-3GalNAca1-3(Fuca1-2)Galb1-4GlcNAcb-Sp0	94	7	7	14.0127979	0.00039351
46	Neu5Aca2-3(6S)Galb1-4GlcNAcb-Sp8	61	18	29	3.4037975	0.02117151
607	Neu5Aca2-3Galb1-4GlcNAcb1-3Galb1-4GlcNAcb1-3Galb1-4GlcNAcb1-2Mana1-6(Neu5Aca2-3Galb1-4GlcNAcb1-3Galb1-4GlcNAcb1-3Galb1-4GlcNAcb1-2Mana1-3)Manb1-4GlcNAcb1-4GlcNAcb-Sp12	60	17	28	3.59145108	0.01849255
603	Neu5Aca2-3Galb1-4GlcNAcb1-3Galb1-4GlcNAcb1-2Mana1-6(Neu5Aca2-3Galb1-4GlcNAcb1-3Galb1-4GlcNAcb1-2Mana1-3)Manb1-4GlcNAcb1-4GlcNAcb-Sp12	60	13	21	4.67896627	0.00922184
237	Neu5Aca2-3GalNAcb1-4GlcNAcb-Sp0	58	21	36	2.75330858	0.03527319
450	Gala1-3(Fuca1-2)Galb1-4GlcNAcb1-6(Gala1-3(Fuca1-2)Galb1-4GlcNAcb1-3)GalNAc-Sp14	48	17	35	2.83924971	0.03284357
362	Fuca1-2Galb1-4(Fuca1-3)GlcNAcb1-2Mana1-6(Fuca1-2Galb1-4(Fuca1-3)GlcNAcb1-2Mana1-3)Manb1-4GlcNAcb1-4GlcNAb-Sp20	43	12	28	3.54099562	0.01916821
328	Neu5,9Ac2a2-3Galb1-4GlcNAcb-Sp0	41	7	17	5.76655319	0.00518281
